# Measuring Post-Partum Haemorrhage in Low-Resource Settings: The Diagnostic Validity of Weighed Blood Loss versus Quantitative Changes in Hemoglobin

**DOI:** 10.1371/journal.pone.0152408

**Published:** 2016-04-06

**Authors:** Esther Cathyln Atukunda, Godfrey Rwambuka Mugyenyi, Celestino Obua, Elly Bronney Atuhumuza, Nicholas Musinguzi, Yarine Fajardo Tornes, Amon Ganaafa Agaba, Mark Jacob Siedner

**Affiliations:** 1 Mbarara University of Science and Technology, Mbarara, Uganda; 2 Department of Medicine and Center for Global Health, Massachusetts General Hospital, Boston, Massachusetts, United States of America; 3 Harvard Medical School, Boston, Massachusetts; Royal Tropical Institute, NETHERLANDS

## Abstract

**Background:**

Accurate estimation of blood loss is central to prompt diagnosis and management of post-partum hemorrhage (PPH), which remains a leading cause of maternal mortality in low-resource countries. In such settings, blood loss is often estimated visually and subjectively by attending health workers, due to inconsistent availability of laboratory infrastructure. We evaluated the diagnostic accuracy of weighed blood loss (WBL) versus changes in peri-partum hemoglobin to detect PPH.

**Methods:**

Data from this analysis were collected as part of a randomized controlled trial comparing oxytocin with misoprostol for PPH (NCT01866241). Blood samples for complete blood count were drawn on admission and again prior to hospital discharge or before blood transfusion. During delivery, women were placed on drapes and had pre-weighed sanitary towels placed around their perineum. Blood was then drained into a calibrated container and the sanitary towels were added to estimate WBL, where each gram of blood was estimated as a milliliter. Sensitivity, specificity, negative and positive predictive values (PPVs) were calculated at various blood volume loss and time combinations, and we fit receiver-operator curves using blood loss at 1, 2, and 24 hours compared to a reference standard of haemoglobin decrease of >10%.

**Results:**

A total of 1,140 women were enrolled in the study, of whom 258 (22.6%) developed PPH, defined as a haemoglobin drop >10%, and 262 (23.0%) had WBL ≥500mL. WBL generally had a poor sensitivity for detection of PPH (<75% for most volume-time combinations). In contrast, the specificity of WBL was high with blood loss ≥ 500mL at 1h and ≥750mL at any time points excluding PPH in over 97% of women. As such, WBL has a high PPV (>85%) in high prevalence settings when WBL exceeds 750mL.

**Conclusion:**

WBL has poor sensitivity but high specificity compared to laboratory-based methods of PPH diagnosis. These characteristics correspond to a high PPV in areas with high PPH prevalence. Although WBL is not useful for excluding PPH, this low-cost, simple and reproducible method is promising as a reasonable method to identify significant PPH in such settings where quantifiable red cell indices are unavailable.

## Introduction

Accurate estimation of blood loss is key to prompt prediction, diagnosis and management of life-threatening post-partum hemorrhage (PPH), which remains a leading cause of maternal morbidity and mortality in low-resource countries (LRC)[1]. Accurate quantification of massive blood loss may prevent hypovolemia, hypotension, tachycardia and consequently organ failure and death as a result of tissue hypoxia. Early diagnosis of hypovolemic shock is therefore of utmost importance especially in settings with large amounts of bleeding. Although hypovolemic shock is detectable by changes in vital signs like tachycardia, hypotension as well as poor tissue oxygenation like pallor, blue lips, and changes in mental status and poor capillary refill, its actual measure and quantification is still vital in prompt diagnosis of PPH.

In LRC settings, blood loss is often estimated by visual estimation by attending health workers (HWs), due to lack of neither adequate skilled labor nor reliable laboratory infrastructure to quantify blood loss. Attempts to standardize this visual inspection method by training HWs to estimate soakage have not been successful, because it has been found to have poor validity and reliability[1,2]. While other methods of blood loss measurement have been better validated, they remain unadopted in LRC due to their complexity and/or cost [2,3]. As such, strategies to simplify blood loss estimation in LRC that allow measurement of blood loss without expensive supplies, complex human resource inputs, or laboratory infrastructure are needed.

Some proposed strategies include calculation of total blood volume[4–7], direct estimation of blood loss using bedpans, fixed-sized gauze pads, calibrated delivery drapes and shallow bed pans[8–13], or transparent collector bags [14]. These direct techniques have been hypothesized to reduce the likelihood of underestimation, leading to improved detection, diagnosis and management of PPH [2,15]. However, most have not been validated against a quantified measurement of blood loss, such as change in peri-partum hemoglobin, which remains the reference standard in high resource settings[16]. Although PPH has been defined as blood loss ≥500 mL after vaginal delivery[17], observed bleeding may not appear abnormal when hemorrhage is internal, as in the case of a vaginal or a broad ligament hematoma. In contrast, change in peri-partum hemoglobin, unlike other methods, detects all forms of blood loss, including hemolysis and internal formation of hematomas[18].

Although direct measurement of blood loss is a potentially cost-effective method to detect PPH in resource limited settings, its diagnostic accuracy remain largely untested. We sought to evaluate the diagnostic accuracy of the weighed blood loss method as compared to quantitative changes in hemoglobin as a reference standard. Our overarching goal was to evaluate if a weighed blood loss method could serve as a valid, low-cost, measure of PPH for use in LRC where laboratory testing is not available.

## Materials and Methods

### Study design and setting

Data from this analysis were collected as part of a randomised controlled trial comparing oxytocin with misoprostol for post-partum haemorrhage (NCT01866241). All study procedures were conducted at the Mbarara Regional Referral Hospital, a publically-funded teaching hospital in rural south-western Uganda serving 10 districts with a population of over 5 million people. The hospital delivers over 10,000 mothers annually and a pre-study review of 9,027 births over 10 months estimated a 33% incidence of maternal PPH.

### Participants and Recruitment

Full study procedures have been described previously[19]. Trained midwife research assistants (MRAs) screened labouring mothers in early active labour on arrival to the prenatal ward. Eligibility criteria were 1) age above 18 years, 2) 38–41 weeks of amenorrhea and 3) anticipated uncomplicated vaginal delivery as assessed by hospital staff. The exclusion criteria included: 1) confirmed foetal death, 2) self-reported maternal heart disease, 3) mothers' current diagnosis of severe malaria or acute bacterial infection, 4) multiple pregnancy, 5) induced or augmented labour, 6) planned Caesarean delivery, 7) ante partum haemorrhage (APH), 8) reported hypersensitivity to prostaglandins and 9) altered cognitive status (ACS) as assessed by the MRAs. MRAs obtained written informed consent from all eligible participants after the birth was determined to likely be uncomplicated vaginal delivery. All consenting participants gave written informed consent, or for those who could not write, a thumbprint was made on the consent form.

### Study Groups

Participants were randomly assigned to 600μg misoprostol sublingually or 10 IU oxytocin intramuscularly within one minute following birth. Delayed cord clamping was preferred and the placenta was delivered by controlled cord traction (CCT) or manually if not delivered 30 minutes postpartum as per Ugandan National Clinical Guidelines (UNCG). Further care was provided by the hospital clinical care team in collaboration with MRAs in accordance with national guidelines which recommends administration of a repeat dose of parenteral oxytocics along with bladder emptying, management of lacerations, and uterine massage if bleeding persists. Mothers were monitored for 24 hours postpartum before discharge.

### Study Measures

Two blood samples for complete blood count (CBC) were drawn 1) immediately after admission and 2) prior to hospital discharge or before the first blood transfusion, for participants who received one. After the baby was born, the amniotic fluid was drained from the plastic sheet immediately. A clean plastic sheet specifically designed and piloted to collect blood for this trial was placed under the mother’s buttocks during and after third stage of labour. Blood collected onto the plastic sheet during delivery was drained into a standard calibrated measuring jar[20] Additionally, mothers were given pre-weighed standard sanitary mops to place in the perineum during the entire postpartum period. These pads were collected, weighed and added onto the volume of blood from the plastic sheet. To improve consistency in estimation of blood loss, standardized electronic scales were used to weigh soiled mops. Mops were weighed hourly for the first six hours, and then every six hours until 24 hours postpartum. At the conclusion of the 24-hour postpartum period, remaining pads were collected, weighed and added to the volume of blood from the plastic sheet. We estimated blood loss as 1 mL per each gram of weighed mop, subtracted from the dry mop weight, as previously described[21]. Study staff who performed laboratory tests and directly measured blood loss were blinded to treatment allocation. The CBC was performed using a Sysmex KX-21N™ Automated Haematology Analyzer (Sysmex Corporation, Kobe, Japan).

### Statistical Analysis

We described demographic and clinical data for the cohort using standard summarization techniques. Our primary aim was to assess the diagnostic validity of weighted blood loss method (WBL) versus quantified blood loss. Our reference standard was hemoglobin (Hb) drop, which is the usual means of measuring blood loss accurately and indirectly, with direct clinical relevance to the risk of myocardial infarction[16]. We considered a dichotomous outcome of Hb drop of >10% as a reasonable definition of PPH as previously used[21]

We assessed the sensitivity and specificity of WBL using the following dichotomous thresholds: a) ≥250mLs, b) ≥500mLs, c) 750mLs, d) ≥ 1000mLs at each of the following time points 1, 2 and 24 hour-post partum.

To describe the performance of the WBL compared to hemoglobin change, we estimated sensitivity and specificity of each volume-time combination (e.g. ≥250mLs at 1 hour, 2 hours, and 24 hours), and fit a receiver operating curve at each time point (i.e. 1 hour, 2 hours, and 24 hours). Finally, we calculated the positive and negative predictive values of each volume-time combination assuming PPH prevalence of 5, 15, and 30% to assess the clinical utility of each measure in various settings. Data analyses were performed using STATA version 12.0 (Statacorp, College Station, Texas, USA).

### Ethical Approval

This study was approved by the Institutional Review Council of Mbarara University of Science and Technology and Uganda National Council of Science and Technology, and registered with clinicaltrials.gov (NCT01866241). All consenting participants gave written informed consent, or for those who could not write, a thumbprint was made on the consent form as approved by the ethics committees. All authors report no conflicts of interest.

## Results

One thousand one hundred and forty participants were randomized and enrolled in the study and had paired haemoglobin and blood loss measured by the WBL. The mean age of participants was 29.5 years (standard deviation [SD] 3.3 years) and mean gestational age was 39.3 weeks (SD 0.8). Mean haemoglobin and hematocrit at admission were 13.2 mg/dL (SD 1.3) and 39.5 (SD 4.0), respectively. Other baseline characteristics are presented in Table 1. In the full cohort, 258 (22.6%) women had post-partum haemorrhage, as defined by a haemoglobin drop of >10%. Using the WBL method, 262(23.0%) of women met criteria for PPH by measured blood loss ≥500 mL and 35 (3.1%) of women met criteria for severe PPH by blood loss ≥1000 mL (Table 2).

**Table 1:** Participant baseline demographic and clinical characteristics.

Characteristics	Mean (SD) or Frequency (%)
Mean age (years)	29.5 (3.3)
Educational attainment less than secondary, n (%)	713 (62.5)
Mean birth weight (kilograms)	3.2 (1.5)
Mean gestational age (weeks)	39.3 (0.8)
Mean Hb admission(g/decilitre)	13.2 (1.3)
Mean HCT admission	39.5 (4.0)
Pre-delivery Hb (g/dl) <12, n(%)	161 (14.1)
Parity, n (%)	
1	468 (41.1)
2–4	559 (49.1)
≥5	111 (9.8)
Perineal tear, n (%)	139 (12.2)
Episiotomy, n (%)	321 (28.2)
History of PPH*, n (%)	131 (19.5)
Prenatal visits (n = 1132), n (%)	
0	1 (0.2)
1–3 visits	180 (16.0)
>3 visits	950 (83.9)
History of home birth*, n (%)	269 (40.1)

Hb: haemoglobin; HCT: Hematocrit; PPH: Post-partum haemorrhage

*Excludes primigravid mothers

**Table 2:** Measurement of PPH by haemoglobin drop and measured blood loss.

Measurement of Post-Partum Haemorrhage	Mean (SD) or Frequency (%)
Haemoglobin change from pre to post-partum (mean, SD)	1.0 (1.0)
Haemoglobin decrease >10% (n, %)	258 (22.6)
Measured blood loss ≥ 500mL (n, %)	
At 1 hour	90 (7.9)
At 2 hours	146 (12.8)
At 24 hours	262 (23.0)
Measured blood loss ≥1000mL (n, %)	
At 1 hour	21 (1.8)
At 2 hours	32 (2.8)
At 24 hours	35 (3.1)

mL: millilitres

**Fig 1:**
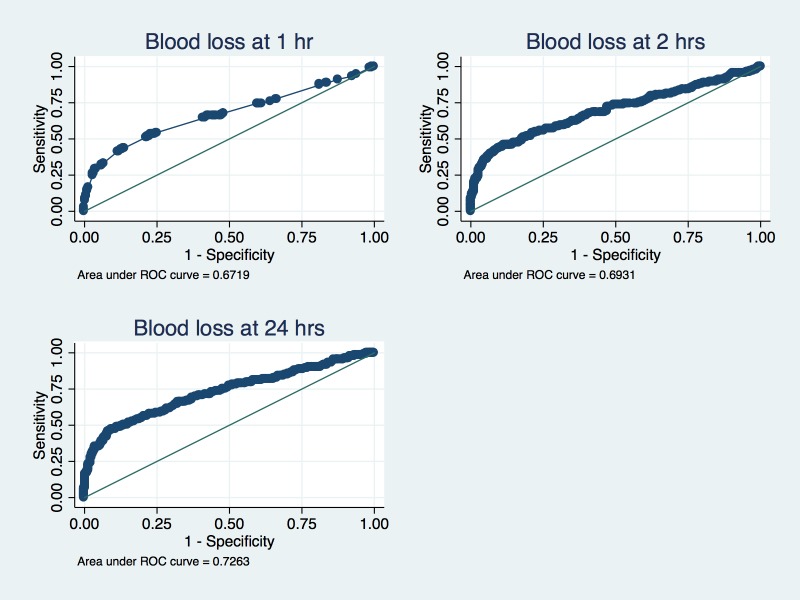
ROC curves comparing measured blood loss and Hb drop (standard) at different time points.

Measured blood loss by the WBL generally had a poor sensitivity for detection of PPH compared to a reference standard of a haemoglobin drop >10%. Aside from blood loss of ≥250mL at 24 hours, no other time point and mop weight combination achieved sensitivity greater than 75% (Table 3). In contrast, the specificity of mop weight measurements was generally high, such that the specificity of WBL ≥ 500mL at 1 hour, and WBL ≥ 750mL and ≥ 1000mL at 1, 2, or 24 hours was over 95. These associations are depicted on ROC curves comparing measured blood loss and drop in postpartum haemoglobin, which are notable for low sensitivity across all three time points over the distribution of most WBL measurements (Fig 1).

**Table 3:** Sensitivity and specificity of blood loss measurement by weight method at Varying time points and volumes of blood loss, compared to reference standard of >10% drop in haemoglobin.

Time of Blood Loss Weight	Measured Blood Loss	Sensitivity (%)		Specificity (%)		Correctly classified (%)
1 hour	≥250mL	129/252	51.2 (45.0–57.4)	699/888	78.7 (76.0–81.4)	72.6
	≥500mL	63/252	25.0 (19.6–30.4)	863/888	97.2 (96.1–98.3)	81.7
	≥750mL	23/252	9.1 (5.6–12.7)	886/888	99.8 (99.5–100)	79.7
	≥1,000mL	19/252	7.5 (4.3–10.8)	886/888	99.8 (99.5–100.1)	79.5
2 hours	≥250mL	188/252	74.6 (69.2–70.0)	387/888	44.0 (40.7–47.2)	50.4
	≥500mL	95/252	37.3 (31.3–43.3)	836/888	94.1 (92.6–95.7)	81.2
	≥750mL	34/252	13.5 (9.3–17.7)	879/888	99.0 (98.3–99.6)	80.2
	≥1000mL	29/252	11.5 (7.6–15.5)	885/888	99.7 (99.3–100.0)	80.2
24 hours	≥250mL	243/252	96.4 (94.1–98.7)	81/888	9.2 (7.3–11.2)	28.4
	≥500mL	129/252	51.2 (45.0–57.4)	755/888	85.0 (82.7–87.4)	77.4
	≥750mL	73/252	29.0 (23.4–34.6)	865/888	97.4 (96.4–98.5)	82.3
	≥1000mL	32/252	12.7 (8.6–16.8)	886/888	99.7 (99.5–100.1)	80.5

We examined the clinical utility of the WBL by assessing negative and positive predictive values (PPV) of each weight volume -time point combination at PPH prevalence rates of 5, 15and 30%. These results suggested that the WBL has low utility in low PPH prevalence settings. For example in a setting with 5% PPH prevalence, the NPV would be approximately 95% (which is the expected rate of a negative test without using any diagnostic test) at all blood loss volume-time combinations, and the positive predictive value never exceeds 70% (Table 4). In contrast, the WBL appears to have more promise as a diagnostic technique in higher prevalence settings, due to its greater positive predictive value at certain blood loss volume-time point combinations. For example, women with WBL ≥750mL at 1, 2, or 24 hours are likely to have a haemoglobin drop >10% (PPV>85%). Nonetheless, since NPVs do not achieve values higher than 86% at any volume-time combination, the WBL does not appear to be effective at identifying women with peri-partum bleeding.

**Table 4:** Negative and positive predictive values of blood loss measurement by weight method versus a reference standard of >10% drop in haemoglobin.

Time of Blood Loss Weight	Measured Blood Loss	5% Prevalence of PPH		15% Prevalence of PPH		30% Prevalence of PPH	
		NPV	PPV	NPV	PPV	NPV	PPV
1 hour	≥250mL	96.8%	11.2%	90.1%	29.8%	79.0%	50.8%
	≥500mL	96.1%	31.8%	88.0%	61.0%	75.2%	79.2%
	≥750mL	95.4%	67.6%	86.2%	87.5%	71.9%	94.5%
	≥1,000mL	95.4%	63.3%	85.9%	85.3%	71.6%	93.4%
2 hours	≥250mL	97.0%	6.5%	90.7%	18.9%	80.0%	36.2%
	≥500mL	96.6%	25.3%	89.5%	53.2%	77.9%	73.4%
	≥750mL	95.6%	41.3%	86.6%	70.2%	72.8%	85.1%
	≥1000mL	95.5%	64.1%	86.5%	85.7%	72.4%	93.6%
24 hours	≥250mL	98.0%	5.3%	93.5%	15.8%	85.6%	31.3%
	≥500mL	97.1%	15.2%	90.8%	37.6%	80.3%	59.4%
	≥750mL	96.3%	37.1%	88.6%	66.4%	76.2%	85.0%
	≥1000mL	95.6%	66.3%	86.6%	86.8%	72.7%	94.1%

PPV: positive predictive value; NPV: negative predictive value

## Discussion

### Main findings

We conducted a post-hoc analysis of a randomized controlled trial of women undergoing uncomplicated vaginal deliveries at a publically-funded regional referral hospital in south-western Uganda to assess the diagnostic accuracy of WBL, compared to change in peri-partum haemoglobin. There are three principle findings from this study. First, the WBL generally has a poor sensitivity for detection of PPH when compared to a peri-partum haemoglobin drop >10%. This is evidenced by the fact that the sensitivity of weighed blood loss failed to detect 25% or more cases of PPH in all women aside from those with ≥ 250mL at 24 hours postpartum, at which point the corresponding specificity was only 5%. As such, our results suggested that WBL will miss an important minority of PPH cases and that no single time-volume threshold can be used to ensure an accurate diagnosis.

Second, in contrast, the specificity of the WBL was generally high, such that multiple volume-time combinations had specificities over 99%. For example, less than 1% of women with WBL ≥750mL at 1 or 2 hours, or with WBL ≥1000mL at any time point was free from PPH, as measured by a haemoglobin drop of >10%. The low rate of false positives at these blood-volume-time combinations makes WBL a promising diagnostic tool for detecting PPH when blood loss exceeds750 mL at less than 2 hours or if it exceeds 1,000 mL at any time.

Lastly, we found the highest utility of the WBL method was observed in high PPH prevalence settings. In low prevalence settings, the combination of a rare PPH outcome and poor sensitivity of the method gives it an overall low utility. For example in areas with a 5% prevalence of PPH, the NPV of weighed blood loss is always approximately 95% (which it would be without any diagnostic test because 5% of women would be expected to have the outcome) and the PPV never exceeds 70%. In contrast, the high specificity and PPV (>90%) of the WBL method for blood loss thresholds of ≥750 at 1 hour postpartum and for ≥1,000 mL at any time point make it a reasonable option as a means of identifying PPH in certain settings where blood cell indices cannot be promptly accessed, such as in the case of rural or home deliveries. Taken together, our results suggest that the WBL method could serve as a valuable “rule-in” test in resource-limited settings where prevalence of PPH is high, but should not be used alone to reassure clinical staff that women have not had a significant bleeding event to cause a serious complication.

Prior studies have had mixed results in comparing the accuracy of measured blood loss to quantified methods of blood loss. While Patel and colleagues found a positive correlation between measured blood loss and other laboratory method of measuring blood loss by photo spectrometry [12], their study was limited by a relatively small sample size (n = 10). Other studies have reported promising correlations between measured blood loss and laboratory methods that quantify blood loss using absorbent paper or sanitary pads and photometric measurement of alkaline hematin [22]. A notable exception is a study by Tourne and colleagues, who compared red cell indices to direct weight of a transparent collector plastic bag placed under the pelvis of mothers just after delivery[14]. Their results were consistent with our study, in that they reported a low sensitivity (38.8%) and high specificity (96%) for weighed blood loss of ≥500mL at approximately 1 hour postpartum. However, that study used a strict criterion of determining PPH, defined as a fall of hematocrit more than 10 points or by a fall of haemoglobin by more than 3g/dL, measured 3 days postpartum as a reference standard.

### Strengths and Limitations

Our study had a number of strengths. We used a quantified reference standard that objectively considers all routes of blood loss, including haemolysis and internal bleeding. We also used standardized techniques to measure blood loss in all study participants with calibrated scales and sanitary mops. The mops, sheets, and scales used for the study were locally available and thus potentially reproducible in similar settings. Lastly, we had a large enough sample size (>1,000 participants) to thoroughly evaluate the WBL method with relatively narrow precision. There were also limitations to this work. Most importantly, we found clumping in blood loss estimates at both 400 and 500mL, likely due to rounding by study staff, which might misrepresent true blood loss in a sub-set of women. However, this form of clumping should only affect estimates at the 500mL threshold. Secondly, accurate estimation of blood loss through weighing is dependent on diversion of other fluid from the mops and collector bags. Although failure to do so might lead to mis-estimation of true blood loss[16], this limitation is likely to be present in non-research settings, so might be an accurate assessment of the technique in actual clinical conditions.

## Conclusion

Early detection of PPH through actual measurement of blood loss and clinical assessment of the patient is crucial to prevent birth complications, including hemorrhagic shock and disseminated intravascular coagulation. Unfortunately, many validated methods for measuring blood loss are costly and unavailable in low-resource settings, and particularly in rural areas in much of sub-Saharan Africa[23]. As such, low-cost alternatives that can be implemented in such settings could serve as useful tools in preventing complications of PPH. We evaluated WBL, a low-cost, reproducible method of PPH estimation in rural Uganda. We found the method to be of low sensitivity, but high specificity, with correspondingly high PPVs in areas with high PPH prevalence. In summary, the WBL, coupled with close clinical assessment has good promise as a low-cost, simple and reasonable alternative method to identify PPH in such settings where quantifiable red cell indices are unavailable, but is not sensitive enough to exclude it. Future research should explore its feasibility and acceptability to detect PPH in non-research settings.

## Supporting Information

S1 Approval Institutional Review Committee (IRC) Approval. (DOC)

S2 Approval Uganda National Council of Science and Technology (UNCST) Approval. (PDF)

S1 Data Dataset. (CSV)

S1 Text Trial Protocol. (PDF)
