# Supplementary figures and images for: Measuring Post-Partum Haemorrhage in Low-Resource Settings: The Diagnostic Validity of Weighed Blood Loss versus Quantitative Changes in Hemoglobin
Source: PLoS One. 2016 Apr 6;11(4):e0152408. doi: 10.1371/journal.pone.0152408 (PMC4822885; doi:10.1371/journal.pone.0152408)

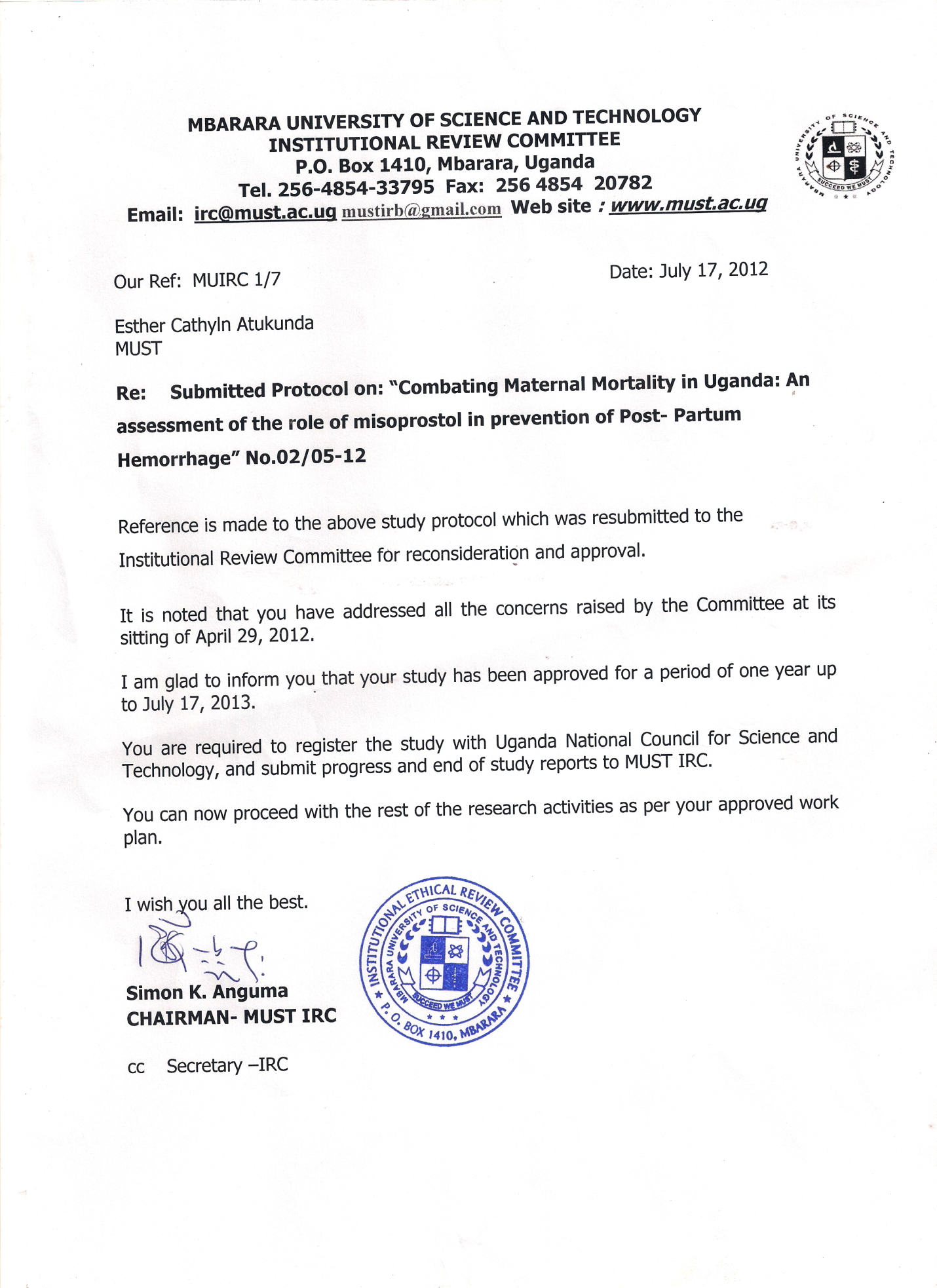

Supplement: S1 Approval — (DOC) [file pone.0152408.s001.doc]
