# Supplementary material for: Measuring Post-Partum Haemorrhage in Low-Resource Settings: The Diagnostic Validity of Weighed Blood Loss versus Quantitative Changes in Hemoglobin
Source: PLoS One. 2016 Apr 6;11(4):e0152408. doi: 10.1371/journal.pone.0152408 (PMC4822885; doi:10.1371/journal.pone.0152408)
Supplement: S2 Approval — (PDF) [file pone.0152408.s002.pdf]

Uganda National Council for Science and Technology

(Established by Act of Parliament of the Republic of Uganda)

UNCST

Our Ref: HS 1245

23rd October 2012

Ms. Esther C. Atukunda
Mbarara University of Science and Technology
P.O Box 1410
Mbarara

Dear Ms. Atukunda,

RE: RESEARCH PROJECT, "COMBATING MATERNAL MORTALITY IN UGANDA: AND ASSESSMENT OF THE ROLE OF MISOPROSTOL IN PREVENTION OF POST-PARTUM HEMORRHAGE, DATED JUNE 2012"

This is to inform you that the Uganda National Council for Science and Technology (UNCST) approved the above research proposal on **10th September 2012.** The approval will expire on **10th September 2013.** If it is necessary to continue with the research beyond the expiry date, a request for continuation should be made in writing to the Executive Secretary, UNCST.

Any problems of a serious nature related to the execution of your research project should be brought to the attention of the UNCST, and any changes to the research protocol should not be implemented without UNCST's approval except when necessary to eliminate apparent immediate hazards to the research participant(s).

This letter also serves as proof of UNCST approval and as a reminder for you to submit to UNCST timely progress reports and a final report on completion of the research project.

Yours sincerely,

Winfred Badanga
for: Executive Secretary
UGANDA NATIONAL COUNCIL FOR SCIENCE AND TECHNOLOGY

Generated by CamScanner from intsig.com
