# Supplementary material for: Measuring Post-Partum Haemorrhage in Low-Resource Settings: The Diagnostic Validity of Weighed Blood Loss versus Quantitative Changes in Hemoglobin
Source: PLoS One. 2016 Apr 6;11(4):e0152408. doi: 10.1371/journal.pone.0152408 (PMC4822885; doi:10.1371/journal.pone.0152408)
Supplement: S1 Text — (PDF) [file pone.0152408.s004.pdf]

FOR OFFICE USE ONLY	Date of submission	Date considered	Approval granted?
Application No. *(Yr/No)* 02/05-12	25/[illegible]/2012	29/04/2012	~~yes~~ / no
Signatures			(Chair) for Amon G. Agaba

INSTITUTIONAL ETHICAL REVIEW COMMITTEE
MBARARA UNIVERSITY OF SCIENCE AND TECHNOLOGY
SUCCEED WE MUST
P.O. BOX 1410, MBARARA

THIS FORM MUST BE TYPEWRITTEN

MBARARA UNIVERSITY OF SCIENCE AND TECHNOLOGY

APPLICATION FORM FOR MUST INSTITUTIONAL REVIEW COMMITTEE APPROVAL

ALL QUESTIONS MUST BE ANSWERED. ANY FORM STATING "SEE PROTOCOL" WILL BE RETURNED. (This form must stand complete in itself).

PLEASE PROVIDE COPIES OF THIS FORM AND THE ORIGINAL PROPOSAL AS STATED IN THE GUIDELINE

AS FAR AS POSSIBLE YOU SHOULD RESTRICT ALL ENTRIES TO THE SPACE PROVIDED ON THIS FORM
Please use a typing font that is easily distinguishable from the questions of the form
NB This form is available on diskette from the Research Support Office

NAME OF APPLICANT: Esther Atukunda Cathyln, Mbarara University of Science and Technology

Have you submitted this proposal to the Mbarara University, Institutional Ethical Review Committee before?

No	Yes	Date and outcome:
	YES	

If you are re-submitting a proposal, please emphasize how the proposal has been amended in the light of previous recommendations from the MUST, Faculty of Medicine Research & Ethics Committee or Institutional Committee

ISSUES RAISED BY THE IRC COMMITTEE THAT SAT ON APRIL 29TH 2012 have been dully addressed (see response letter attached)

If this proposal is for work that will go towards a higher degree (e.g. M.Med or PhD), please state name and Department of Supervisor(s):

1. **Associate Professor Amon G. Agaba,** MD, PhD. Head of Department Pharmacology and Therapeutics
2. **Prof. Celestino Obua, MD, PhD.** Deputy Principal Makerere University College of Health Sciences
3. **Dr. Marc Twagirumukiza,** MD, PhD (Senior lecturer/ consultant physician, Ghent University) physician, clinical pharmacologist. Post-doctoral Scientist AMASA Project.

SECTION A

FOR OFFICE USE ONLY	Date of submission	Date considered	Approval granted?
Application No. *(Yr/No)* 02/05-12	25/[illegible]	29/04/2012	~~yes~~ / no
Signatures [signature]			[signature] (Chair) for Amon G. Agaba

INSTITUTIONAL ETHICAL REVIEW COMMITTEE MBARARA UNIVERSITY OF SCIENCE AND TECHNOLOGY SUCCEED WE MUST P.O. BOX 1410, MBARARA

THIS FORM MUST BE TYPEWRITTEN

MBARARA UNIVERSITY OF SCIENCE AND TECHNOLOGY

APPLICATION FORM FOR MUST INSTITUTIONAL REVIEW COMMITTEE APPROVAL

ALL QUESTIONS MUST BE ANSWERED. ANY FORM STATING "SEE PROTOCOL" WILL BE RETURNED. (This form must stand complete in itself).

PLEASE PROVIDE COPIES OF THIS FORM AND THE ORIGINAL PROPOSAL AS STATED IN THE GUIDELINE

AS FAR AS POSSIBLE YOU SHOULD RESTRICT ALL ENTRIES TO THE SPACE PROVIDED ON THIS FORM
Please use a typing font that is easily distinguishable from the questions of the form
NB This form is available on diskette from the Research Support Office

NAME OF APPLICANT: Esther Atukunda Cathyln, Mbarara University of Science and Technology

Have you submitted this proposal to the Mbarara University, Institutional Ethical Review Committee before?

No	Yes	Date and outcome:
	YES	

If you are re-submitting a proposal, please emphasize how the proposal has been amended in the light of previous recommendations from the MUST, Faculty of Medicine Research & Ethics Committee or Institutional Committee

ISSUES RAISED BY THE IRC COMMITTEE THAT SAT ON APRIL 29TH 2012 have been dully addressed (see response letter attached)

If this proposal is for work that will go towards a higher degree (e.g. M.Med or PhD), please state name and Department of Supervisor(s):

1. **Associate Professor Amon G. Agaba,** MD, PhD. Head of Department Pharmacology and Therapeutics
2. **Prof. Celestino Obua, MD, PhD.** Deputy Principal Makerere University College of Health Sciences
3. **Dr. Marc Twagirumukiza,** MD, PhD (Senior lecturer/ consultant physician, Ghent University) physician, clinical pharmacologist. Post-doctoral Scientist AMASA Project.

SECTION A

SECTION A
STUDY OUTLINE

A.1 TITLE OF Study: Combating Maternal Mortality in Uganda: An assessment of the role of misoprostol in Prevention of Post-Partum Hemorrhage.

A.2 SUMMARY

Explain why this study is being conducted, using lay terminology.

Guidance note:

Please convey what you think is the importance of the research and WHY it is being carried out.

Of the more than 500,000 peri-partum maternal deaths worldwide, 98% occur in developing countries (Hill et al, 2007; Khan et al, 2006.The maternal mortality rate in Uganda is among the highest in the world at 435 for every 100,000 women (UDHS,2011) claiming the lives of over 5,500 mothers annually. Twenty-five percent of these deaths in Uganda occur due to postpartum hemorrhage (PPH) within 24 hours of delivery (MoH, 2012)

Oxytocin, a hormone that stimulates uterine contractions and helps control bleeding immediately after birth is the standard of care for prevention of PPH during the third stage of labor(WHO,2010). The use of oxytocin, however, is limited by a number of factors including a requirement for trained personnel, cold chain storage, sterile syringes and needles for administration leading to inconsistent or no availability in some settings (Prata et al, 2013). Misoprostol, a synthetic prostaglandin with uterotonic properties has been proposed as an alternative strategy for prevention of PPH, because it is administered orally, has a long shelf life, and is stable at room temperature (Tang et al,2002). A sub-lingual formulation is also available and enables a more rapid onset of action and greater bioavailability since it avoids first-pass metabolism (Katzung et al, 2007). Despite these advantages, sublingual misoprostol is not clearly the preferred agent because of insufficient information on its comparative efficacy with oxytocin for prevention of PPH in the management of third stage of labor (Tuncalp et al, 2012; Gulmezoglu et al, 2007).

INSTITUTIONAL ETHICAL REVIEW COMMITTEE
MBARARA UNIVERSITY OF SCIENCE AND TECHNOLOGY
SUCCEED WE MUST
P. O. BOX 1410, MBARARA

In Uganda, up to 97.2% of health facilities expected to provide basic Emergency Obstetric Care do not offer them due to unavailability of electricity, laboratory services, trained staff (midwives) and medicines among others (Mbonye et al., 2006; 2007). Only 46% of the overall deliveries occur in presence of a skilled health worker in contrast to 95% attending at least one antenatal care (PATH, 2010; VSI, 2010; USAID, 2008; WHO, 1997) yet deaths due to PPH occurs within 24 hours following delivery due to excessive blood loss (Ronsmans& Graham, 2006; Khan et al, 2006; Li et al, 1996). Other authors have documented the share of births attended by skilled health personnel to be as low as 29% among the poorest within developing countries (USAID, 2008; UDHS, 2006). Majority of these women give birth at home (Knippenberg, 2005) with an estimated 18 million deliveries in Sub-Saharan Africa assisted by Traditional Birth Attendants (TBAs), a family member or alone (Lawn, 2005).The use of misoprostol on the other hand, which is cheap, does not require refrigeration and its heat stable properties has been shown to be effective in the prevention and management of PPH when used with or without controlled cord traction or uterine massage (Derman et al, 2006), a possibility of giving vast lifesaving hope in low resource setting.

We generally intend to document the role of misoprostol in management of third stage of labor in Uganda. In order to help resolve the question of efficacy in prevention of PPH, we intend to perform a double-blind, double-dummy randomized controlled trial of sublingual misoprostol versus oxytocin at Mbarara regional referral hospital, Uganda. In this trial, we hypothesize that sublingual misoprostol will be non-inferior to oxytocin because of its additional advantage of rapid onset of action and greater bioavailability compared to oral route.

A.3 OBJECTIVES

List the major objectives/hypothesis, which have governed your choice of study design

INSTITUTIONAL ETHICAL REVIEW COMMITTEE
MBARARA UNIVERSITY OF SCIENCE AND TECHNOLOGY
SUCCEED WE MUST
P. O. BOX 1410, MBARARA

General objective

To examine the role of sublingual misoprostol 600µg in the management of third stage of labor in Uganda

Specific objectives

1. To assess the efficacy of sublingual misoprostol 600µg, a prostaglandin E1 analogue as an alternative first-line uterotonic in prevention of PPH in Mbarara Regional Referral Hospital (MRRH), South Western Uganda
   a) To compare maternal blood loss within 24 hours after delivery between misoprostol and oxytocin groups of mothers delivering at MRRH.
   b) To compare the pre and post delivery changes in hematocrit and hemoglobin levels in the Misoprostol and oxytocin groups of delivering mothers at MRRH.
   c) To determine the proportion of women who require additional therapeutic procedures or uterotonics in third stage of labor for treatment of PPH in the Misoprostol group.
   d) To compare length of third stage of labour between the misoprostol and oxytocin groups of mothers delivering from MRRH
   e) To determine the proportion of women with retained placenta between the two groups.
   f) To compare the proportion of women requiring and getting blood transfusion between misoprostol and oxytocin groups.

2. To assess the safety (shivering, fever, headache, diarrhea, after pains, death, nausea/vomiting) of sublingual misoprostol 600µg as an alternative prophylactic uterotonic to oxytocin in the management of third stage of labor to prevent PPH.

Hypothesis

Ho: 10 IU Oxytocin is significantly better than sublingual misoprostol 600µg in management of third stage of labor

H1: Sublingual misoprostol 600µg is non- inferior to 10 IU oxytocin and will not be more than 6% worse [than 10 IU oxytocin] in management of third stage of labor

INSTITUTIONAL ETHICAL REVIEW COMMITTEE
MBARARA UNIVERSITY OF SCIENCE AND TECHNOLOGY
SUCCEED WE MUST
P.O. BOX 1410, MBARARA

A.4 METHODOLOGY

Outline how you intend to achieve the objectives of the study.

Guidance notes:

For each objective/hypothesis:-

- *define the target population*
- *describe how the sample(s) is(are) to be recruited from the target population(s)*

Even if the main thrust of the research is biomedical, the rationale behind your use of social science methods (e.g. patient interviews) should be clear.

describe how the sample(s) is(are) to be recruited from the target population(s)

INSTITUTIONAL ETHICAL REVIEW COMMITTEE
MBARARA UNIVERSITY OF SCIENCE AND TECHNOLOGY
SUCCEED WE MUST
P. O. BOX 1410, MBARARA

Objective 1 and 2 will be achieved through a proposed non-inferiority trial of sublingual misoprostol 600µg. Following arrival of the laboring mothers at the maternity ward, mothers will be assessed and admitted. History will be reviewed to identify mothers eligible for vaginal delivery. Only mothers 38-41 weeks of amenorrhea admitted and anticipating vaginal delivery at MRRH will be eligible to participate in the study. Mothers with IUFD, hypertensive disorders, cardiac or coronary disease, inflammatory bowel disease, elective or previous Caesarean Section, gestation age < 38 weeks of amenorrhea and hypersensitivity to prostaglandins will be excluded from this study due to their associated risks to PPH (Chien, 1999; Combs et al, 1991).

Randomization

Consecutive numbers ranging from 001 to 1140 will be computer generated to indicate the two main groups into which mothers can be randomly assigned and enrolled. These consecutive numbers will be labeled on top of the opaque envelopes. Once eligibility of a mother is confirmed, they will be invited to participate in the study. The aim of the study and details of the procedures to be involved in the trial for both oxytocin and misoprostol groups, potential side effects and therapeutic benefits will be explained before randomization occurs. Once the mothers consent to participate in the study, a study number will be allocated by the midwife research assistant (**who will be recruited to work on the ward mainly for this study**) by taking the next in a series of similar opaque envelopes provided to conceal allocation of groups. These opaque envelopes will be labeled with computer-generated list of numbers with group allocation (either COHORT or Comparison Groups) of the mothers randomized in blocks of 10. Midwife research assistants will be blinded to the treatment group allocation and will open the envelope to prepare the treatments in the envelope provided and pre-packed by an independent pharmacy technician when vaginal delivery is eminent. This administration will be within one minute after delivery.

A first blood sample for complete blood count (CBC) will then be drawn immediately after admission and consenting of the eligible mothers to document Hemoglobin (Hb), hematocrit levels, MCV, blood group & cross matching (to document baseline values and assess need for transfusion). The second blood sample will be drawn at discharge or before blood transfusion to estimate pre-transfusion levels. The socio-demographic data and obstetric characteristics will also be documented. The patient will then be monitored for progress of labor. In case a consented mother ends up in theatre for caesarean section, the time and indication will be documented and excluded. A total of 1140 adult mothers will be enrolled for this study

INSTITUTIONAL ETHICAL REVIEW COMMITTEE
MBARARA UNIVERSITY OF SCIENCE AND TECHNOLOGY
SUCCEED WE MUST
P. O. BOX 1410, MBARARA

A.5 PARTICIPANTS

Please provide the following information on the participants with/from whom you expect to be collecting data:

A.5.1 Age / Sex: (please enter the expected number in each of the boxes)

	Neonates (<28 days)	Infants (1-11 months)	Young children (1-9 years)	Adolescents (10-17 years)	Adults (18 yrs & above)
Males					
Females					1140

Guidance notes:-
This age/sex breakdown helps convey how vulnerable the participants will be
If you are unable to give precise figures, state estimates and give an explanatory sentence in the space below

A.5.2 What specific measures are in place to take into account women of childbearing age?
Guidance notes:
women may have different responses to disease processes
The developing foetus may be particularly vulnerable in intervention trials

The study involves administration of a uterotonic agent (misoprostol or oxytocin) after the delivery of the baby. The drugs are not also known to circulate into the lactating milk, nor do they pose a big risk to these women

A.5.3 Describe how and where the participants are to be recruited?
Guidance notes:
This is distinct from the statistical sampling method described in A.4. You should outline the procedures for recruitment of each group of participants, include details on:
- *the setting (e.g. Country, Town, District, on the ward, out-patient department ,in the home)*
- *inclusion and exclusion criteria for selection, if relevant (e.g. "Women of child-bearing age will be excluded")*

The Randomized Controlled Trial (RCT) study will purposively be carried out in the Maternity ward of Mbarara Regional Referral Hospital (MRRH), rural south western Uganda which also doubles as a Mbarara University Teaching Hospital for the faculty of medicine. The hospital is located in Kamukuzi Division, Mbarara Municipality along 30° 20E' and 31°20'E longitude and 1°30'S and 0°30'N Latitude (DISH, 2011http://www.ugandadish.org/). The regional referral hospital receives mothers from different tribal and socio-demographic locations in Uganda. Though inadequate, the hospital is equipped with trained staff, midwives, and obstetricians able to offer emergency obstetric care (EMOC) facilities. The Hospital also has good blood transfusion services and theatre for additional therapeutic procedures that may be required to treat PPH. Mothers received in this hospital may therefore represent different mothers from various social and demographic backgrounds.

INSTITUTIONAL ETHICAL REVIEW COMMITTEE
MBARARA UNIVERSITY OF SCIENCE AND TECHNOLOGY
SUCCEED WE MUST
P. O. BOX 1410, MBARARA

Mothers with IUFD, hypertensive disorders, cardiac or coronary disease, inflammatory bowel disease, elective or previous Caesarean Section, gestation age < 38 weeks of amenorrhea and hypersensitivity to prostaglandins will be excluded from this study due to their associated risks to PPH (Chien, 1999; Combs et al, 1991). Mothers will be consented during early stages of active labor (before 6 cms of cervical dilation) to give ample time for mothers to think through the consent process and avoid feeling coarsed. The consented mothers will then be randomly assigned different treatment groups. These mothers will be blinded to the study group allocated.

Intervention (COHORT) group

The intervention group will be given sublingual misoprostol 600µg as part of the routinely done active management of third stage of labor within one minute of delivery. In this group, sublingual misoprostol 600µg will be administered as a prophylactic uterotonic agent. Delayed cord clamping will also be preferred due to documented benefits to the baby (Farrar 2009a; RCOG, 2009; Mc Donald, 2008; Van Rheenan, 2007; Hutton, 2007; Chaparro, 2006; Ceriani et al, 2006; Rabe, 2004). Blinding will also be done by administration of 1 ml of normal saline to mimic oxytocin injection.

Control (COMPARISON) group

The comparison group on the other hand will involve the standard/ gold procedure in the management of third stage of labor. Mothers will be given oxytocin 10 IU as the prophylactic uterotonic agent within one minute following delivery of the baby. This time according to Tharakan and Jha (2008) is more effective and safe compared to injection after delivery of placenta. This group will also receive a placebo taken sublingually to blind for misoprostol. This group will be the control group. Delayed cord clamping will also be preferred.

In all the two groups, the placenta will be delivered manually if not delivered after 30 minutes. In case bleeding persists, additional uterotonic agents, bladder emptying, expulsion of clots, blood transfusion or surgical procedures like hysterectomy will be used to manage PPH and this will be documented for all the groups.

Same sublingual route of administration will be preferred due to its pharmacodynamics properties including; rapid onset of action, greater bioavailability

INSTITUTIONAL ETHICAL REVIEW COMMITTEE
MBARARA UNIVERSITY OF SCIENCE AND TECHNOLOGY
SUCCEED WE MUST
P. O. BOX 1410, MBARARA

and prolonged activity (Tang et al, 2002; Andolina, 2003; Hofmeyr, 2005; Khan, 2003; Abdel-Aleem, 2003). Similar misoprostol doses will also be maintained to avoid variations in plasma therapeutic levels (Mousa and Alfirevic, 2009, Tang et al, 2002). Single doses of recommended 600µg misoprostol will be used as opposed to repeated doses (FIGO/ICM, 2006).

A.5.4 Please justify your choice of sample size (as described in A.4)

The two main research questions guiding this particular trial were to assess whether sublingual 600µg misoprostol, with an advantage of not requiring skilled administration and special storage was no worse (non-inferior) than 10 IU oxytocin in prevention of PPH. The trial is also to assess whether sublingual misoprostol 600µg is reasonably efficacious and a feasible alternative to oxytocin in the active management of thirds stage of labor especially in resource-limited settings. The primary outcome of this trial will be maternal blood loss $\geq$500 mL within 24 hours postpartum.

In a superiority trial, one's null hypothesis is that the two groups are equal and your alternative hypothesis (or your main hypothesis) would be that one intervention is better (superior) than the other, in this case oxytocin (clinically/ technically, oxytocin has been preferred to misoprostol). In an equivalence trial (also known as a two-sided non-inferiority trial), your null hypothesis is that one arm is better than the other, and your alternative hypothesis is that they are equal. In a non-inferiority trial, your null is that one arm is better than the other and your alternative hypothesis is that the alternative intervention (with particular advantages- misoprostol) is not worse or non-inferior to the standard (oxytocin). A pre-defined margin of non-inferiority (Δ) has to be stated since it is almost impossible to prove exact equality (Gulmezoglu et al, 2009; Piaggio et al, 2009; Lesaffre, 2007). This Δ defines the upper bound (Δ_{NI}) and alternative treatment will be considered non-inferior to the standard if the 95% CI Δ lies left of Δ_{NI}. Unlike in the superiority trials, non-inferiority trials are unpopular reported using p-values but (two-sided) 95% CI (Lesaffre, 2007). Non-inferiority trial therefore requires rejection of a null hypothesis (H_0: $\Delta > \Delta_{NI}$) and prove the alternative (H_1: $\Delta < \Delta_{NI}$). Alternatively, $p<0.025$ is considered as significant to prove that the alternative treatment is not worse/ non-inferior based on predefined Δ_{NI} (D'Agostino et al, 2006).

INSTITUTIONAL ETHICAL REVIEW COMMITTEE
MBARARA UNIVERSITY OF SCIENCE AND TECHNOLOGY
SUCCEED WE MUST
P. O. BOX 1410, MBARARA

To determine the inferiority margin (Δ_{NI}), the effect of standard treatment (oxytocin) was compared and its benefit over intervention treatment (misoprostol) in prevention of PPH ($\geq$ 500 mls). Several Cochrane reviews and well designed trials that had blood loss as an outcome were considered. A 6% difference in blood loss of $\geq$ 500 mls was therefore considered to be confident that misoprostol is a reasonable alternative to oxytocin and of vital benefit compared to no intervention This is within the percent window of +/- 10% to be confident that misoprostol can be superior, inferior, non-inferior or equal to oxytocin.

For studies intending to test a non-inferiority hypothesis, the recommended equation for sample size determination (WHO, 2010; Feinstein, 2002; Fleiss, 1980)

$$n \text{ (for each group)} = \frac{2x\,[sd]^2}{\delta^2} \times (Z_\alpha + Z_\beta)^2$$

For a test of proportions, the sd= $p_1q_1 + p_2q_2$ (where p_1= probability in standard group and p_2= probability in intervention group. Q=1-p)

$$n = \frac{2x\,[p_1(1-p_1) + p_2(1-p_2)]^2}{\delta^2} \times (Z_\alpha + Z_\beta)^2$$

Prophylactic oxytocin (standard treatment) reduces the rate of blood loss > 500mls by 50% compared to no uterotonics at all/ placebo (RR 0.50, 95% CI 0.43 to 0.59) (Cotter et al, 2007, Elbourne et al, 2001; Predniville et al, 2001)

Oral misoprostol significantly reduces the rate of acute PPH (>500mls of blood loss) by 47% compared to placebo (RR 0.53, 95% CI 0.39-0.74, p<.0001) (Derman et al, 2006)

A multi-site study significantly documented proportions of 20% for PPH in the misoprostol arm compared to 14% in the oxytocin arm for post-partum hemorrhage ($\geq$ 500 mls) (Gulmezoglu et al, 2001)

P_1= 0.20; P_2= 0.14

delta (δ) = 6% (0.06)

A level of confidence interval 95% (two sided) will be considered and therefore Z_{crit} = 1.96

A statistical power of 90% will be considered for Z

Given a predicted incidence of 14% PPH in mothers treated with prophylactic oxytocin and 28% for those who receive no intervention (placebo), a delta of 6% is within the limits

INSTITUTIONAL ETHICAL REVIEW COMMITTEE
MBARARA UNIVERSITY OF SCIENCE AND TECHNOLOGY
SUCCEED WE MUST
P. O. BOX 1410, MBARARA

required to be confident that misoprostol is a reasonable alternative to oxytocin and of vital benefit compared to no intervention. A total of **1140** mothers will therefore be needed to achieve a 90% power (two sided) to say that misoprostol is no worse than oxytocin in PPH prevention. Each of the two groups, misoprostol and oxytocin will comprise of 570 mothers.

A.6 PROCEDURES

A.6.1 What procedures or methods will be employed in the collection of data (e.g. patient interviews / focus group discussions / blood sampling / biopsies) and by whom (e.g. experienced facilitator / social scientist / teacher/ qualified doctor / nurse, auxiliary, etc.)?

Attach additional sheets if necessary.

**Method**	**To be carried out by:**
Patient interviews	Midwife Research assistants (4)
Blood samples	Midwife Research Assistant or Laboratory Technician (2)
Observations	Midwife Research assistants
Preparation of treatments to be given	Pharmacy technician (2)
Data entry	Qualified data clerks (2)

A.6.2 State the extent to which the procedures to be used are a part of usual clinical management (if appropriate).

- Observations proposed are routinely done to monitor progress of labor
- Blood samples are sometimes requested for Hb, grouping and cross matching in case there is an indication for possible PPH

A.6.3 Please indicate that the persons identified in A.6.1 are competent to carry out these procedures. List any training of staff that may be required prior to commencement of the study.

The project personnel will include qualified midwives to collect blood samples and data (both interviews and observations), a laboratory technician to collect blood samples, a pharmacy technician to prepare the proposed treatments and a data clerk to double check and enter data

A.7 ANALYSIS

INSTITUTIONAL ETHICAL REVIEW COMMITTEE
MBARARA UNIVERSITY OF SCIENCE AND TECHNOLOGY
SUCCEED WE MUST
P. O. BOX 1410, MBARARA

A.7.1 What are the major statistical (or other) methods that you intend to use to analyse the data to fulfil each of the objectives/hypothesis stated in A.3

Data Preparation

All data will be cross checked for completeness before discharge of mothers. Data will be coded and entered independently into access database and exported into STATA version 12·0 (Statacorp, College Station, Texas, USA) for statistical analysis.

3.4.2 Data Analysis Plan

Data analysis will be by intention-to-treat (ITT) consisting of all mothers randomized and thus supposed to be treated (Piaggio et al, 2006; Lesaffre, 2008). Frequencies will be obtained for each variable on Laboratory and individual data to establish proportions or missing values. Different variables will be explored for normal distribution. The proportions of blood loss in oxytocin and misoprostol groups will be compared with t- tests. Selected demographic, clinical, pre and peri-natal factors will be compared for differences and similarities in the two groups (misoprostol and oxytocin). Crude relative risks will be derived. Relative risks with 95% confidence Interval (two tailed) will be calculated and used to compare the effect of treatment outcomes in both groups. We will also compare continuous outcomes using student t-tests. Univariate analysis will be done for blood loss $\geq$ 500mls and for results whose p value exceeds 0.10, a multivariate analysis will be done to rule out confounding factors. Relative risks of the acute, severe PPH other secondary outcomes and maternal side effects will also calculated and compared across groups.

3.4.3 Data Reporting

Results will be analyzed and presented as per the current CONSORT guidelines (Piagio et al,2006).The proportions will be presented as percentages and their confidence Intervals will be presented at 95% (two-tailed) at the power of 95%. Two-tailed statistical analysis will be used and statistical significance will be defined at level of $p \leq 0.05$. If the lower limit of the 95% confidence interval lies to the right of the non-inferiority margin (Δ) the experimental intervention will be regarded "**inferior**". If results lie left of Δ, it will be concluded as **non-inferior** with regard to that pre-stated margin Δ. If the results are inconclusive regarding the non-inferiority hypothesis and the whole 95% confidence interval lies to the right of zero, it will be concluded that the misoprostol is **significantly worse** according to a superiority hypothesis.

INSTITUTIONAL ETHICAL REVIEW COMMITTEE
MBARARA UNIVERSITY OF SCIENCE AND TECHNOLOGY
SUCCEED WE MUST
P. O. BOX 1410, MBARARA

A.8 QUALITY ASSURANCE

A.8.1 What procedures are in place to ensure the quality of the data?

Guidance notes:
For qualitative data (for example) what procedures will be used to check translations or compare data obtained from different sources?
For quantitative data (for example) how will transcription errors be minimised?
Give some detail on how methods are going to be piloted, if appropriate

- An instrument to collect the required data has been adapted from the tools used by other researchers in other sites.
- Computer-generated numbers will be used to randomize mothers into this study. These numbers will be in opaque envelopes to blind the observers on the group to which the next mother will be randomized and observed. The Investigator or midwife researchers/ observers will not have prior knowledge of the treatment allocated to the next patient to avoid bias into the random allocation process.
- Double blinding will be done by administration of 1 ml of normal saline or water for injection to the misoprostol group to mimic oxytocin injection. **All the inactive agents (dummy oxytocin/ water for injection and placebo for misoprostol) will be packaged to exactly resemble the actual drugs themselves to ensure effective double blinding.** The comparison group on the other hand involving the standard/ gold oxytocin as a prophylactic uterotonic agent will also receive a placebo taken sublingually to blind for misoprostol.
- Details of the aim and procedures to be involved in the trial for both oxytocin and misoprostol groups, potential side effects and therapeutic benefits will be explained to the eligible patients on admission by the midwife researcher before consent and randomization occurs. Once the mothers consent to participate in the study, they will be monitored for labor and opaque envelopes containing computer-generated consecutive numbers from 001-1140 will then be opened once vaginal delivery is deemed inevitable.

INSTITUTIONAL ETHICAL REVIEW COMMITTEE
MBARARA UNIVERSITY OF SCIENCE AND TECHNOLOGY
SUCCEED WE MUST
P. O. BOX 1410, MBARARA

- Improved routines at the labor ward will be targeted and all mothers on the ward regardless of the intervention will be treated similarly. A 'PPH Trolley' will be freely provided at all times in the labor ward to improve availability and accessibility of these uterotonics.
- All blood samples for complete blood count will be labeled properly using patient random study numbers on the laboratory request form, interview guides and observation forms. Laboratory tests and examinations will be done by two specific laboratory technicians to ensure consistency. All samples will be stored in the same laboratory to ensure consistency and accuracy. Blood samples will be randomly selected and tested in another laboratory for quality checks, consistency and accuracy.
- Sublingual route of administration of misoprostol will be preferred due to its pharmacodynamics properties including rapid onset of action, greater bioavailability and prolonged activity (Hofmeyr, 2005; Khan, 2003; Abdel-Aleem, 2003). Similar misoprostol doses will also be maintained and administered once to avoid variations in plasma therapeutic levels (Mousa and Alfirevic, 2009)
- After the baby has been born, the amniotic fluid will be drained immediately and a plastic sheet will placed under the mother's buttocks to collect blood during and after third stage of labor. After the delivery of the placenta, mothers will be asked to put on similar pre-weighed sized pads for the next 24 hours.
- Electronic weighing scales will be standardized and some bloody mops will be sampled and weighed using other alternative scales from time to time to ensure consistency and accuracy
- An internationally recognized brand of misoprostol (cytotec) or its equivalent from a company with International Good Manufacturing Practice will be used for this study
- Four midwives will be **recruited** and **trained** as observers to increase accuracy and consistency in estimation and recording of blood loss plus other treatment outcomes. They will be assisted by the labor staff and these outcome assessors will be blinded to the interventions and hypotheses of this study. To minimize observer bias and facilitate patient and observer randomization, each

INSTITUTIONAL ETHICAL REVIEW COMMITTEE
MBARARA UNIVERSITY OF SCIENCE AND TECHNOLOGY
SUCCEED WE MUST
P. O. BOX 1410, MBARARA

research assistant will be assigned the even or odd study numbers alternating every time an eligible mother is identified. Senior Obstetricians will supervise the trial, collection and recording of blood and other treatment outcomes on maternity ward. Observations will be done from the onset of 3rd stage of labor to 24 hours after delivery.

- Expired blood will be obtained and used in training on estimating spillage on floor, tables and garments in case it occurs. These spills on garments, floor and tables will be minimized. The bloody pads for the next 24 hours will be measured soon after delivery to avoid drying and evaporation.
- **A safety monitoring committee incorporating the independent members with expertise in Obstetrics and Statistics will be constituted to ensure safety of participating mothers.** Four safety checks will be done at 25% recruitment, 50%, 75% and at the end of data collection.
- All laboratory tests will be independently done by qualified staff in Mbarara University of Science and Technology Research laboratory. Similar calibrated containers and 1g pre-weighed cotton pads will be utilized to collect and estimate blood loss. **A spare laboratory technician will be hired and on hand to back up or handle laboratory tests at all times**

A.9 DISSEMINATION OF RESULTS
Please outline what plans you have for dissemination of results.
Guidance notes:
Where possible a mechanism should be in place to inform study participants of the outcomes of the study.
It is important that study findings are made known to local services / policy makers before they are discussed (e.g.) at international scientific meetings

The study strategy for knowledge management and dissemination will be done by all investigators. Particularly, the dissemination of results for this study is motivated by both the academic and social impact, with a focus on the practical benefits for the key maternal health in Uganda and Low-resource settings.

The following points are highlights of the approach:

- Local meetings with key stakeholders in health policy, pharmaceutical sector, and other sectors of civil society and government
- Presentations at institutional, national and international conferences
- Specially tailored open-source materials (such as policy briefs, articles for magazines that reach policymakers or presentations to policymakers)

INSTITUTIONAL ETHICAL REVIEW COMMITTEE
MBARARA UNIVERSITY OF SCIENCE AND TECHNOLOGY
SUCCEED WE MUST
P.O. BOX 1410, MBARARA

- Submission and publication of research findings to international high quality peer-reviewed journals

SECTION B

CONSEQUENCES FOR THE LOCAL COMMUNITY / ENVIRONMENT AND PARTICIPANTS

B.1 Outline the potential adverse effects, discomfort or risks that may result from the study in the following areas:

B.1.1 Participants

Guidance note:

In addition to the physical effects of tissue sampling (for example blood sampling) it should be borne in mind that interviews and focus group discussions may sometimes trigger painful or distressing memories (e.g. questions about sexual practice or the death of a child)

1. Most if not all questions that will be asked have been classified 'not sensitive or personal'. However, the participants may feel some anxiety answering some of them. In this case, they may choose not to answer some questions they feel uncomfortable

2. There is the risk of discomfort when blood is being drawn. There is also a small risk of bruising or infection. However. no more than 4mls of blood will be drawn for each of the two blood draws described. A participant may also refuse blood draws at any time.
3. There is a risk of bleeding following child birth for either of the study groups (oxytocin or misoprostol). In case this happens, routine procedures will be followed to give the participant additional therapeutic medications or procedures to treat or prevent further bleeding. Trained health professionals will also be available during and after delivery to treat this bleeding and answer any further questions

INSTITUTIONAL ETHICAL REVIEW COMMITTEE
MBARARA UNIVERSITY OF SCIENCE AND TECHNOLOGY
SUCCEED WE MUST
P. O. BOX 1410, MBARARA

B.1.2 Investigators

Guidance notes:

Include here (for example)

- *the biomedical risks to investigators (including local staff) involved in tissue sampling (e.g. Hepatitis B, HIV)*
- *the psychological consequences for social science investigators exposed to narratives of violence or severe grief*
- *the risks from the environment (e.g. in a war zone)*

There are anticipated risks to Hepatitis B or HIV especially since the study is targeting measuring blood loss as its primary outcome.

B.1.3 Members of the public

We do not expect any adverse effects, discomfort or risks to members of the public.

B.2 Outline what steps will be taken to minimise the adverse effects, discomfort or risks described above.

B.2.1 For participants

Guidance notes:

In biomedical research, appropriate use of anaesthesia prior to procedures (for example) is important.
For social science research it may be necessary to ensure that counselling services are available for those who re-live traumatic experiences through (for example) an in depth interview.

- Most if not all questions that will be asked have been classified 'not sensitive or personal'. However, the participants may feel some anxiety answering some of them. In this case, they may choose not to answer some questions they feel uncomfortable
- The risk of discomfort, bruising or infection when blood is being drawn will be minimized by engaging highly trained personnel in phlebotomy. Also, no more than 4mls of blood will be drawn for each of the two blood draws described and a participant may also refuse blood draws at any time.
- There risk of bleeding following child birth for either of the study groups (oxytocin or misoprostol) will be managed by following routine procedures to give the participant additional therapeutic medications, bladder emptying, expulsion of clots or procedures to treat or prevent further bleeding. Trained health professionals will also be available during and after delivery to treat this bleeding and answer any further questions
- The research assistant will not discuss any information obtained from the participant with anyone other than the research and health care team

[P

INSTITUTIONAL ETHICAL REVIEW COMMITTEE
MBARARA UNIVERSITY OF SCIENCE AND TECHNOLOGY
SUCCEED WE MUST
P. O. BOX 1410, MBARARA

- Refusing to participate in this study at any time will not affect healthcare services that a participant would ideally obtain from the ward
-

B.2.2 For investigators

Guidance notes:
Where the research may involve adverse experiences for investigators (see B.3.2), de-briefing / support meetings may be important.

> Protective gear like aprons and gloves will be readily provided and availed at all times to prevent this risk for all data collectors and the laboratory staff doing blood tests.
> Procedures to obtain post exposure prophylaxis will also be streamlined and services availed to all staff at all time through the routine hospital policy.
> Training on infection control will be done and emphasized for all the staff involved in this study.

B.2.3 For members of the public

> ADVERSE EXPERIENCES ARE NOT EXPECTED

B.3 CONSEQUENCES FOR LOCAL HEALTH SERVICES

B.3.1 What demands will this research place on local health services?

Guidance notes:
For example, how much of a nurse's usual work time will be taken up in acting as an interpreter for an outside investigator?

> During the study, which is expected to last a maximum period of about 2 years, the midwife RAs may require to use the routine admission room, labor suite and post-natal ward to monitor progress of labor and document observations. They may also take up some available working space and use the available refrigerator to keep treatments. The midwives may also require assistance from fellow midwives in taking required peri or post natal observations and carrying out deliveries. Midwives speaking the local language will however be recruited for this study

B.3.2 Detail how the design of the research project takes into account the demands described in 3.1.

Guidance notes:
Disruption to routine services should be kept to a minimum.

> The study will not disrupt the routine services. Oxytocin has been used as a standard prophylactic uterotonic agent in PPH management and the NMS has recently started to supply misoprostol as an alternative used during oxytocin stockouts or unavailable skilled personnel

B.4 CONFIDENTIALITY AND PRIVACY

INSTITUTIONAL ETHICAL REVIEW COMMITTEE
MBARARA UNIVERSITY OF SCIENCE AND TECHNOLOGY
SUCCEED WE MUST
P. O. BOX 1410, MBARARA

B.4.1 What steps will be taken to ensure privacy and confidentiality for participants?

1. All Research Assistants will undertake a course on Research on Human subjects before the study commences
2. Participants in this study will be ensured of their privacy. The research assistant will not discuss any information obtained from the participant anyone other than the research and health care team
3. Research data will be collected on a hard copy marked with an anonymized study identification number. This data will be entered and maintained in an electronic format on the computer that will be password protected at all times. The hard copies will be kept in a lockable cabin for safety and privacy. Researcher will take every extra precaution to prevent this from happening by always excluding any information to identify you personally.
4. All research records will be handled as confidentially as possible. All research records will be coded with an anonymised study ID so that no person outside the study group can identify the participant. No individual identities will be used in any reports or publications that result from this study. No individual identities will be included in all the data shared with other researchers. Laboratory results will be given to the participant and will only be shared with the medical team involved in her care at the ward.
5. Consent will be sought from the respondents before enrollment into the study

B.5 INFORMED CONSENT

B.5.1 Information given to participants:

Please indicate what you will tell the participants in simple language. The purpose of the study, type of questions that will be asked, and procedure or treatment which will be applied should be described and reference should be made to possible side effects, discomfort, complications and/or benefits. If a specific consent form is available, please attach.

1. It will be made clear to the participant that he/she is free to decline to participate or to withdraw at any time without affecting healthcare services obtain from the ward or any other disadvantage or prejudice. The consent form has been attached.

5.2 Outline who will deliver the above information and how?

The information will be delivered by a trained midwife Research Assistants of the study.

- They will share information about the study with the participants, i.e, introduce themselves, explain the purpose and type of the research to be carried out, and invite the participants to join the study, on a voluntary basis. This will be done in earlier stages of labor preferably before 6 cm cervical dilatation when labor pains are anticipated to be mild to moderate. This will give the participants ample time to think through the consent process and make an informed decision without coercion.
- The RAs will also explain the procedures, risks and benefits, ensure confidentiality, dissemination of results, and whom to contact later, for further questions.
- They will provide a certificate of Consent to the participants for their signatures

B.5.3 Please indicate how consent will be obtained, given local circumstances.

Guidance notes:

In some societies, the concept of giving consent on an individual basis is unfamiliar. It may be necessary to obtain consent both at community and individual level.

Obtaining consent from minors requires both consent from the guardian and, where possible, the minor.

Consent will be sought on an individual basis and only adult mothers will be approached for possible participation in the study.

B.5.4 Are any inducements to be offered to either participants or the individuals who will be recruiting them? (e.g. improved patient care / cash) (please tick appropriate box)

Yes	No
	√

B.5.5 If yes, please give details:

N/A

B.5.6 Outline any hidden constraints to consent.

Guidance notes:

Examples where hidden constraints may be important include:

- *situations where participants are employees of the investigator*
- *patients who may feel their care could be compromised if they do not consent to research initiated by their carers.*

- Patients may feel obliged or a responsibility to participate in the study fearing any sort of bias to be offered routine care in case they do not consent. However, efforts will be put to explain to the participant that participation is purely voluntary, with no disadvantages in case they chose to withdraw at whatever stage of the study. The participants will also be contacted at an early stage of labor when labor pains are assumed to be mild to moderate ($\leq$ 6 cms of cervical dilatation) to allow ample time to think through the consent process, the benefits and risks and consult their next of kins regarding their voluntary participation.

INSTITUTIONAL ETHICAL REVIEW COMMITTEE
MBARARA UNIVERSITY OF SCIENCE AND TECHNOLOGY
SUCCEED WE MUST
P. O. BOX 1410, MBARARA

SECTION C
RESPONSIBILITY

C.1 Litigation:

In respect of any litigation which may result from this research
a) Who will provide compensation?

Misoprostol 600ug and lower doses have been documented to be safe when used for PPH prevention and or treatment. Particular litigation is not envisaged in the overall AMASA research framework or this particular study. However, this issue will be brought to the attention and review of the Data and safety Monitoring committee in case safety issues do arise.

This data and safety monitoring committee will constitute the senior staff on the maternity ward to ensure safety of participating mothers. Four safety checks will be done at 25% recruitment, 50%, 75% and at the end of data collection.

(Please provide documentary evidence where appropriate.)

b) What insurance arrangements have been made by the applicant and his/her delegated assistants?

As in C.1 a. above
(Please ensure that any professional indemnity insurance is logged with the Director's office)

INSTITUTIONAL ETHICAL REVIEW COMMITTEE
MBARARA UNIVERSITY OF SCIENCE AND TECHNOLOGY
SUCCEED WE MUST
P. O. BOX 1410, MBARARA

C.2 **DECLARATION: TO BE SIGNED BY MAIN APPLICANT**

- I confirm that the details of this proposal are a true representation of the research to be undertaken.

- I will ensure that the research does not deviate from the protocol described.

- If significant protocol amendments are required as the research progresses, I will submit these to the Mbarara University Faculty Medicine Research Ethics Committee for approval.

- Where an appropriate mechanism exists, I undertake to seek additional local Ethical Approval in the country(ies) where the research is to be carried out.

I expect the study to commence on (Date): 1st August, 2012 and be completed by (Date): 1st August 2014.

.

18/06/2012

Signed Date

SECTION D
APPROVALS

D.1 List research team and all collaborators.
(Please include all overseas collaborators and give their affiliations, qualifications and role in the study).

Name	Designation
Assoc. Prof. Pamela K. Mbabazi (MA, MSc, PhD)	Deputy Vice Chancellor,, AMASA Site Lead, Mbarara University of Science and Technology (MUST); **Promoter**
Assoc. Prof. Amon Ganaafa Agaba (MBChB, PhD)	Head of Department of Pharmacology & Therapeutics, AMASA Site Co-Lead-MUST; **Local Supervisor**
Esther Atukunda, MPH (Leeds)	**PhD Researcher**

International supervisors

1. **Prof. Celestino Obua, MD, PhD.** Deputy Principal Makerere University College of Health Sciences.
2. **Dr. Marc Twagirumukiza,** MD, PhD (Ghent University) physician, clinical pharmacologist. Post-doctoral Scientist AMASA Project.

INSTITUTIONAL ETHICAL REVIEW COMMITTEE
MBARARA UNIVERSITY OF SCIENCE AND TECHNOLOGY
SUCCEED WE MUST
P. O. BOX 1410, MBARARA

Data Safety and Monitoring Board and stopping rules

1. Assoc. Prof Pamela K. Mbabazi, PhD (Mbarara University of Science and Technology
2. Dr. George Wasswa Ssalongo, Mmed Obs/ Gyn, Senior consultant, Mbarara Regional referral hospital
3. Dr. Joseph Ngonzi, Mmed Obs/Gyn, Mbarara University of Science and Technology
4. Mr. Elly Atuhumuza (Bio-statician), Medical Research Council, Entebbe
5. Prof Celestino Obua, PhD, Deputy Principal, Makerere University College of Health Sciences

This is purely an independent DSMB from Mbarara University, Makerere University, Mbarara Hospital and an independent biostatician working with a reputable research organization in Entebbe. The preliminary results will be reviewed at 50% (570), 75% (855) and completion. The committee will base their decision to stop the trial after consideration of disadvantages of the treatment arm over the conventional/control arm and its likelihood to affect not only maternal clinical outcomes but also practice for both acute and severe PPH. This decision will be based on a two-sided superiority hypothesis.

INSTITUTIONAL ETHICAL REVIEW COMMITTEE
MBARARA UNIVERSITY OF SCIENCE AND TECHNOLOGY
SUCCEED WE MUST
P. O. BOX 1410, MBARARA
